# Supplementary material for: M1 Macrophage-Biomimetic Targeted Nanoparticles Containing Oxygen Self-Supplied Enzyme for Enhancing the Chemotherapy
Source: Pharmaceutics. 2023 Aug 30;15(9):2243. doi: 10.3390/pharmaceutics15092243 (PMC10534656; doi:10.3390/pharmaceutics15092243)
Supplement: Supplementary file 1 [file pharmaceutics-15-02243-s001.zip › pharmaceutics-2544159-supplementary.pdf]

Supplementary Materials: M1 macrophage-biomimetic targeted nanoparticles containing oxygen self-supplied enzyme for enhancing the chemotherapy

Jiayi Zhang, Bing Gu, Shimiao Wu, Lin Liu, Ying Gao, Yucen Yao, Degong Yang, Juan Du, Chunrong Yang

Supplementary information:

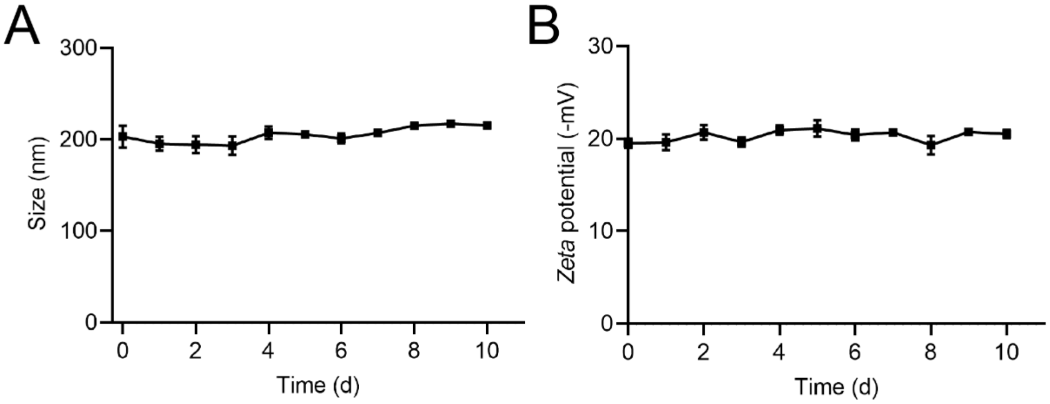

Figure S1. The placement stability of DOX/CAT@PLGA-M1 particle size (A), *zeta* potential (B) (n = 3).
